# Respiratory and Nonrespiratory Diagnoses Associated With Influenza in Hospitalized Adults

**DOI:** 10.1001/jamanetworkopen.2020.1323

**Published:** 2020-03-20

**Authors:** Eric J. Chow, Melissa A. Rolfes, Alissa O’Halloran, Nisha B. Alden, Evan J. Anderson, Nancy M. Bennett, Laurie Billing, Elizabeth Dufort, Pam D. Kirley, Andrea George, Lourdes Irizarry, Sue Kim, Ruth Lynfield, Patricia Ryan, William Schaffner, H. Keipp Talbot, Ann Thomas, Kimberly Yousey-Hindes, Carrie Reed, Shikha Garg

**Affiliations:** 1 Epidemic Intelligence Service, Center for Surveillance, Epidemiology and Laboratory Services, Centers for Disease Control and Prevention, Atlanta, Georgia; 2 Influenza Division, National Center for Immunization and Respiratory Diseases, Centers for Disease Control and Prevention, Atlanta, Georgia; 3 Communicable Disease Branch, Colorado Department of Public Health and Environment, Denver; 4 Departments of Medicine and Pediatrics, Emory University School of Medicine, Atlanta, Georgia; 5 Emerging Infections Program, Atlanta, Georgia; 6 Veterans Affairs Medical Center, Atlanta, Georgia; 7 Department of Medicine, University of Rochester School of Medicine and Dentistry, Rochester, New York; 8 Bureau of Infectious Diseases, Ohio Department of Health, Columbus; 9 New York State Department of Health, Albany; 10 California Emerging Infections Program, Oakland; 11 Salt Lake County Health Department, Salt Lake City, Utah; 12 New Mexico Department of Health, Albuquerque; 13 Communicable Disease Division, Michigan Department of Health and Human Services, Lansing; 14 Minnesota Department of Health, St Paul; 15 Maryland Department of Health, Baltimore; 16 Division of Infectious Disease, Vanderbilt University School of Medicine, Nashville, Tennessee; 17 Oregon Public Health Division, Portland; 18 Connecticut Emerging Infections Program, Yale School of Public Health, New Haven

## Abstract

**Importance:**

Seasonal influenza virus infection is a major cause of morbidity and mortality and may be associated with respiratory and nonrespiratory diagnoses.

**Objective:**

To examine the respiratory and nonrespiratory diagnoses reported for adults hospitalized with laboratory-confirmed influenza between 2010 and 2018 in the United States.

**Design, Setting, and Participants:**

This cross-sectional study used data from the US Influenza Hospitalization Surveillance Network (FluSurv-NET) from October 1 through April 30 of the 2010-2011 through 2017-2018 influenza seasons. FluSurv-NET is a population-based, multicenter surveillance network with a catchment area that represents approximately 9% of the US population. Patients are identified by practitioner-ordered influenza testing. Adults (aged ≥18 years) hospitalized with laboratory-confirmed influenza were included in the study.

**Exposures:**

FluSurv-NET defines laboratory-confirmed influenza as a positive influenza test result by rapid antigen assay, reverse transcription–polymerase chain reaction, direct or indirect fluorescent staining, or viral culture.

**Main Outcomes and Measures:**

Acute respiratory or nonrespiratory diagnoses were defined using *International Classification of Diseases, Ninth Revision, Clinical Modification* (*ICD-9-CM*) and *International Statistical Classification of Diseases and Related Health Problems, 10th Revision (ICD-10)* discharge diagnosis codes. The analysis included calculation of the frequency of acute respiratory and nonrespiratory diagnoses with a descriptive analysis of patient demographic characteristics, underlying medical conditions, and in-hospital outcomes by respiratory and nonrespiratory diagnoses.

**Results:**

Of 89 999 adult patients hospitalized with laboratory-confirmed influenza, 76 649 (median age, 69 years; interquartile range, 55-82 years; 55% female) had full medical record abstraction and at least 1 *ICD* code for an acute diagnosis. In this study, 94.9% of patients had a respiratory diagnosis and 46.5% had a nonrespiratory diagnosis, including 5.1% with only nonrespiratory diagnoses. Pneumonia (36.3%), sepsis (23.3%), and acute kidney injury (20.2%) were the most common acute diagnoses. Fewer patients with only nonrespiratory diagnoses received antiviral therapy for influenza compared with those with respiratory diagnoses (81.4% vs 88.9%; *P* < .001).

**Conclusions and Relevance:**

Nonrespiratory diagnoses occurred frequently among adults hospitalized with influenza, further contributing to the burden of infection in the United States. The findings suggest that during the influenza season, practitioners should consider influenza in their differential diagnosis for patients who present to the hospital with less frequently recognized manifestations and initiate early antiviral treatment for patients with suspected or confirmed infection.

## Key Points

**Question:**

Which types of respiratory and nonrespiratory diagnoses were associated with influenza in hospitalized adult patients since the 2009 influenza pandemic?

**Findings:**

In this cross-sectional analysis of more than 80 000 adults hospitalized with laboratory-confirmed influenza between 2010 and 2018 in the United States, 95% of patients had a respiratory diagnosis, and 46% had a nonrespiratory diagnosis, including 5% with exclusively nonrespiratory diagnoses.

**Meaning:**

Influenza virus infection may be associated with both respiratory and nonrespiratory diagnoses, highlighting the broad scope of influenza burden of disease.

## Teaser

This cross-sectional study examines the respiratory and nonrespiratory diagnoses reported for US adults hospitalized with laboratory-confirmed influenza between 2010 and 2018.

## Introduction

Influenza virus infection generally causes self-limited respiratory symptoms; however, in some patients, illness may be more severe, requiring hospitalization or resulting in death. The Centers for Disease Control and Prevention (CDC) estimates that 140 000 to 810 000 hospitalizations and 12 000 to 61 000 deaths among adult patients are attributable to influenza in the United States annually depending on the season.^1^ Although pneumonia is the most common respiratory complication of influenza,^2,3^ influenza is also known to exacerbate underlying chronic respiratory diseases, including asthma or chronic obstructive pulmonary disease,^4,5^ leading to increased health care use.^6^ Although respiratory diagnoses of seasonal influenza virus infection are well described, less is known about the breadth of nonrespiratory diagnoses.

Nonrespiratory diagnoses of influenza are likely underrecognized^7^; however, previous studies have detailed the association of acute myocardial infarction,^8,9^ seizures and other neurologic manifestations,^10,11^ acute kidney injury,^12^ and sepsis^13,14^ with influenza. Other less common but important diagnoses that have led to hospitalization include encephalitis^7^ and acute myocarditis.^15^

The US Influenza Hospitalization Surveillance Network (FluSurv-NET) conducts population-based surveillance for laboratory-confirmed influenza-associated hospitalizations and represents approximately 9% of the US population.^16^ Previous FluSurv-NET studies in adults^17^ and children^18^ have compared the diagnoses of seasonal influenza before 2009 with those seen during the 2009 H1N1 influenza pandemic, but diagnoses in hospitalized adults have not been extensively detailed using population-based surveillance since that time. This study describes the frequency and variety of respiratory and nonrespiratory diagnoses and outcomes associated with laboratory-confirmed influenza-associated hospitalizations during the influenza seasons from 2010 to 2018.

## Methods

### Study Design, Setting, and Participants

This cross-sectional analysis used data from FluSurv-NET.^16^ Patients included adult residents of the FluSurv-NET catchment area who were 18 years or older and hospitalized with laboratory-confirmed influenza during October 1 through April 30 of the 2010-2011 through 2017-2018 influenza seasons. During these seasons, FluSurv-NET conducted surveillance in select counties in the following US states: California, Colorado, Connecticut, Georgia, Maryland, Michigan, Minnesota, New Mexico, New York, Ohio, Oregon, Tennessee, and Utah. Additional sites were included in the network during specific seasons: Idaho (2010-2011), Iowa (2012-2013), Oklahoma (2010-2011), and Rhode Island (2010-2013). We defined laboratory-confirmed influenza virus infection within 14 days before or 3 days or less after hospital admission based on a positive result of reverse transcription–polymerase chain reaction, rapid antigen assay, direct or indirect fluorescent staining, or viral culture. Testing for influenza was ordered at the discretion of the treating health care practitioner, and no criteria were provided to direct influenza testing. The CDC determined this surveillance project was not human subjects research; therefore, the CDC’s institutional review board approval was not required. The FluSurv-NET sites obtained human study participants and ethics approvals from their respective academic partner and state health department institutional review boards as appropriate. Informed consent was not obtained because data were collected as part of routine public health surveillance (not subject to institutional review board approval). Data were deidentified prior to delivery to the CDC. This study followed the Strengthening the Reporting of Observational Studies in Epidemiology (STROBE) reporting guideline.

### Variables, Data Sources, and Measurement

For each patient, trained surveillance officers using a standardized case reporting form abstracted demographic data (including self-reported race/ethnicity), chronic medical conditions, clinical course and outcomes (length of stay, admission to the intensive care unit [ICU], use of mechanical ventilatory assistance, use of extracorporeal membrane oxygenation, or in-hospital mortality), and discharge summary data from the medical record. We used the first 9 *International Classification of Diseases, Ninth Revision, Clinical Modification* (*ICD-9-CM*)– and *International Statistical Classification of Diseases and Related Health Problems, 10th Revision (ICD-10)*–coded discharge diagnoses as captured by FluSurv-NET to classify each patient’s acute diagnoses during hospitalization. We excluded patients without any documented *ICD* codes. To assess how well influenza and related *ICD* codes are documented among persons hospitalized with confirmed influenza, we calculated the percentage of patients with discharge codes for influenza (*ICD-9-CM* codes 487-488 and *ICD-10* codes J09-J11), pneumonia and influenza (*ICD-9-CM* codes 480-488 and *ICD-10* codes J09-J18), and respiratory and circulatory diagnoses (*ICD-9-CM* codes 460-519 and 390-459 and *ICD-10* codes J00-J99 and I00-I99).

We categorized *ICD* discharge codes as acute diagnoses by the terms *acute, acute on chronic,* or *exacerbation*. For *ICD* codes that did not contain these terms, we further classified diagnoses as acute if they were not known to be chronic conditions (eg, sepsis, bacteremia, rhabdomyolysis, and anaphylaxis). We categorized *ICD* codes into the following acute diagnosis groups: respiratory, neurologic, cardiovascular, endocrine, gastrointestinal, hematologic, acute kidney injury, anaphylaxis, sepsis, bacteremia, and transplant diagnoses. We excluded patients who were not categorized into 1 of these groups from the descriptive analysis that focused on acute diagnoses. A comprehensive list of acute *ICD-9-CM* and *ICD-10* codes and their group classification are given in eTable 1 in the Supplement. Similar to proposed changes for the new *International Statistical Classification of Diseases, 11th Revision (ICD-11)*, we classified cerebrovascular accidents (ischemic and hemorrhagic stroke) under acute neurologic diagnoses.^19,20^

During the 2010-2011 through 2016-2017 seasons, medical record abstraction was completed for all patients through FluSurv-NET. Because of the increased number of influenza-related hospitalizations during the 2017-2018 influenza season, a minimum set of variables was collected for all patients, and a sampling scheme was implemented for more detailed medical record abstraction for patients 50 years or older. Each surveillance site had the option to complete medical record abstraction for 100%, 50%, or 25% random samples of patients 65 years or older and 100% or 50% random samples of those aged 50 to 64 years. All patients younger than 50 years and those of any age who died during their hospitalization had complete medical record abstraction.

### Statistical Analysis

For analysis, we weighted data to reflect the probability of selection using SAS survey procedures (SAS Institute Inc); data from seasons 2010-2011 through 2016-2017 were given a weight of 1, whereas data from 2017-2018 were assigned a weight based on each site’s sampling scheme. We report sample sizes as unweighted numbers. Percentages and median (interquartile range) are reported as weighted values. We compared baseline demographic and clinical characteristics of patients with any acute respiratory diagnoses with those with only acute nonrespiratory diagnoses using the Rao-Scott χ^2^ test or the Fisher exact test for categorical variables and the Wilcoxon Mann-Whitney test for continuous variables. We considered 2-sided *P* < .05 to be statistically significant and analyzed data using SAS software and SAS-Callable SUDAAN software, version 9.4.

## Results

**Figure 1.**
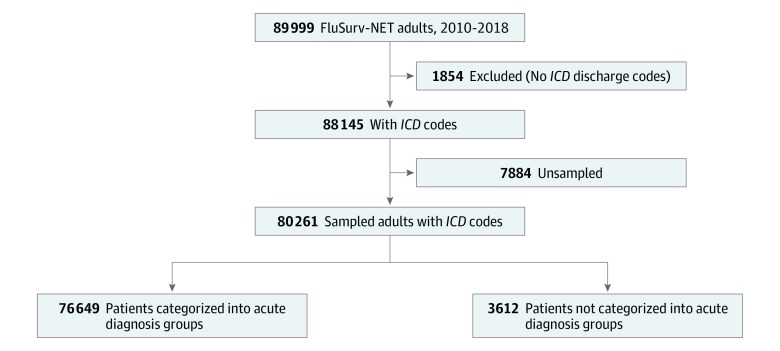
Study Flow Diagram of Adults in the US Influenza Hospitalization Surveillance Network (FluSurv-NET), 2010-2018 *ICD* indicates *International Classification of Diseases*.

During the 2010-2011 through 2017-2018 influenza seasons, 89 999 adults (median age, 69 years; interquartile range, 54-81 years; 55% female) with laboratory-confirmed influenza in the US were captured by FluSurv-NET (Figure 1). We excluded 1854 sampled patients across seasons for which *ICD* codes were not reported. Of the 88 145 adults with at least 1 *ICD* code recorded, 7884 were not sampled for medical record abstraction from the 2017-2018 season. After these exclusions, 80 261 adults were available for analysis. Overall, 81.8% had at least 1 influenza ICD code, 86.2% had a pneumonia and influenza ICD code, and 98.1% had a respiratory and circulatory ICD code (eFigure 2 in the Supplement).

Of the 80 261 adults available for analysis, 76 649 (95.6%) (median age, 69 years; interquartile range, 55-82 years; 55% female) had at least 1 acute diagnosis; 47.3% had received the seasonal influenza vaccine for the corresponding year, and 88.5% had received antiviral treatment for influenza during their hospitalization. Among the 3612 patients who were not categorized into an acute diagnosis group, essential hypertension and unspecified hyperlipidemia were the 2 most common *ICD* codes recorded (eTable 2 in the Supplement). Of the 76 649 adults with an acute diagnosis, 94.9% had at least 1 acute respiratory diagnosis and 46.5% had at least 1 acute nonrespiratory diagnosis, including 5.1% with only acute nonrespiratory diagnoses (eFigure 1 in the Supplement). Patients with at least 1 acute respiratory diagnosis and those with only nonrespiratory diagnoses were similar with respect to age, sex, race/ethnicity, and current season’s influenza vaccination status (Table 1).

**Table 1.** Characteristics of Patients With and Without Acute Respiratory Diagnoses Among Hospitalized Adults With Influenza in the FluSurv-NET, United States, 2010-2018^a^

Characteristic	Patients with respiratory diagnoses (n = 72 997)^b^	Patients without respiratory diagnoses (n = 3652)
Influenza season		
2010-2011	4083 (5.1)	126 (2.9)
2011-2012	1674 (2.1)	37 (0.9)
2012-2013	9201 (11.5)	352 (8.2)
2013-2014	7235 (9.1)	282 (6.6)
2014-2015	13 586 (17.0)	623 (14.5)
2015-2016	6655 (8.4)	329 (7.6)
2016-2017	13 811 (17.3)	913 (21.2)
2017-2018	16 752 (29.5)	990 (38.1)
Age, y		
Overall, median (IQR)	69 (54-82)	70 (57-82)
18-49	14 281 (17.9)	665 (15.5)
50-64	17 360 (23.2)	854 (22.5)
65-74	13 196 (18.8)	693 (20.4)
75-84	12 842 (18.4)	686 (19.5)
≥85	15 318 (21.7)	754 (22.1)
Sex		
Male	32 287 (44.3)	1814 (49.5)
Female	40 710 (55.7)	1838 (50.5)
Race/ethnicity		
Non-Hispanic		
White	45 246 (62.3)	2042 (56.2)
Black	13 075 (17.6)	809 (21.4)
Hispanic	5050 (6.9)	276 (7.4)
Other	9626 (13.2)	525 (15.0)
BMI, No./total No. (%)^c^		
Underweight	2798/65 481 (4.3)	149/3321 (4.6)
Normal	18 987/65 481 (29.2)	1024/3321 (31.0)
Overweight	18 603/65 481 (28.5)	1019/3321 (30.5)
Obesity	18 286/65 481 (27.8)	827/3321 (25.1)
Morbid obesity	6807/65 481 (10.1)	302/3321 (8.8)
Vaccinated in corresponding year		
Yes	34 138 (47.3)	1716 (48.5)
No	29 307 (39.2)	1424 (37.9)
Not documented	9552 (13.5)	512 (13.6)
Tobacco use history, No./total No. (%)		
Current^d^	14 281/67 545 (20.4)	547/3492 (14.9)
Previous^e^	20 495/67 545 (30.9)	1103/3492 (32.7)
Never used or unknown^d^	32 769/67 545 (48.6)	1842/3492 (52.4)
Underlying medical conditions		
No medical history	5535 (7.5)	203 (5.3)
Chronic disease		
Neurologic	17 890 (24.6)	1097 (29.9)
Respiratory tract	31 612 (43.3)	939 (26.2)
Cardiovascular	28 840 (40.0)	1814 (51.2)
Metabolic	30 324 (42.0)	1886 (51.5)
Renal	13 810 (19.4)	1214 (33.3)
Hepatic disease^d^	2670 (3.9)	229 (6.5)
Immunosuppressive^d^	12 364 (16.8)	708 (19.4)
Hematologic^d^	3302 (4.2)	232 (5.8)
Pregnant, among women of childbearing age, No./total No. (%)	1714/8279 (20.7)	21/318 (5.3)
Received influenza antiviral treatment during hospitalization, No/total No. (%)	64 373/72 887 (88.9)	2907/3645 (81.4)
Time from respiratory symptom onset to admission, median (IQR), d^d^	2.3 (1.1-4.1)	2.3 (1.0-4.3)

Abbreviations: BMI, body mass index (calculated as weight in kilograms divided by height in meters squared); FluSurv-NET, US Influenza Hospitalization Surveillance Network; IQR, interquartile range.

^a^ Data are presented as number (percentage) of patients unless otherwise indicated. Numbers are unweighted values, and percentages are weighted values. Percentages are column percentages.

^b^ Includes patients with only acute respiratory diagnoses or those with both respiratory and nonrespiratory diagnoses.

^c^ Underweight measured as BMI less than 18.5; normal weight, 18.5 to 24.9; overweight, 25.0 to 29.9; obesity, 30.0 to 39.9; and morbid obesity, 40 and greater.

^d^ From 2011 to 2018.

^e^ From 2012 to 2018.

Of the 94.9% of patients with at least 1 acute respiratory diagnosis, 43.3% had underlying respiratory comorbidities, and 51.3% were current or former tobacco users (Table 1). Patients with only acute nonrespiratory diagnoses had a significantly higher frequency of underlying medical comorbidities compared with patients with respiratory diagnoses, including neurologic (29.9% vs 24.6%; *P* < .001), cardiovascular (51.2% vs 40.0%; *P* < .001), metabolic (51.5% vs 42.0%; *P* < .001), renal (33.3% vs 19.4%; *P* < .001), hepatic (6.5% vs 3.9%; *P* < .001), immunosuppressive (19.4% vs 16.8%; *P* < .001), and hematologic (5.8% vs 4.2%; *P* < .001) diagnoses. Despite similar timing from respiratory symptom onset to admission for those with only nonrespiratory diagnoses (median, 2.3 days; interquartile range, 1.0-4.3 days) and those with a respiratory diagnosis (median, 2.3 days; interquartile range, 1.1-4.1 days), patients with only nonrespiratory diagnoses were less likely to be receive antiviral treatment compared with patients with a respiratory diagnosis (81.4% vs 89.9%; *P* < .001).

**Figure 2.**
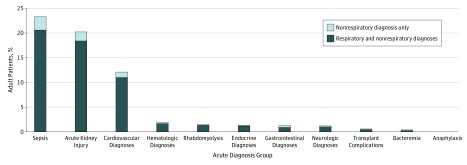
Nonrespiratory Diagnoses Among 76 649 Adults Hospitalized With Laboratory-Confirmed Influenza in the US Influenza Hospitalization Surveillance Network (FluSurv-NET), United States, 2010-2018 Diagnoses are not mutually exclusive.

The most common acute respiratory diagnoses were influenza with other respiratory manifestations (56.1%) and pneumonia (36.3%) (eTable 3 in the Supplement). The most common acute nonrespiratory diagnoses overall were sepsis (23.3%), acute kidney injury (20.2%), and acute cardiovascular events (12.1%) (Figure 2); these acute diagnoses were also the most common among those with exclusively acute nonrespiratory diagnoses. Of note, there were no diagnoses of Reye syndrome in the adult population.

Among patients with acute respiratory and nonrespiratory diagnoses, influenza A viruses were detected in 80% and influenza B virus in 20% of patients (Table 2). A similar percentage of patients with influenza A and B virus infections were diagnosed with an acute respiratory diagnosis, acute kidney injury, sepsis, and acute cardiovascular events (Table 2 and eTable 3 in the Supplement). Of patients with influenza A virus infection, 43.7% had subtyping performed, and 74.5% were subtyped as A(H3N2); there was a greater frequency of cardiac diagnoses among those with A(H3N2) (13.4%) compared with those with A(H1N1)pdm09 (9.6%). However, this difference was not statistically significant when stratifying by age groups (*P* > .05 across all age groups).

**Table 2.** Hospitalizations With Acute Diagnoses by Influenza Type and Subtype in FluSurv-NET, United States, 2010-2018^a^

Acute Diagnosis	Patients, No. (%)				
	Total (N = 76 649)	Influenza A (n = 61 862)	Influenza B (n = 14 303)	Influenza A	
				(H1N1)pdm09 (n = 7243)	H3N2 (n = 20 194)
Respiratory tract diagnosis	72 997 (94.9)	59 052 (95.2)	13 489 (93.7)	6974 (96.3)	19 214 (95.0)
Neurologic diagnosis	939 (1.2)	739 (1.2)	192 (1.3)	85 (1.2)	241 (1.2)
Cardiovascular event	9046 (12.1)	7319 (12.1)	1671 (12.0)	690 (9.6)	2642 (13.4)
Endocrine diagnosis	1143 (1.4)	883 (1.4)	251 (1.6)	121 (1.6)	280 (1.4)
Gastrointestinal tract diagnosis	1038 (1.3)	792 (1.3)	229 (1.6)	140 (1.9)	248 (1.2)
Hematologic diagnosis	1488 (1.9)	1196 (1.9)	279 (1.8)	178 (2.4)	357 (1.8)
Other diagnoses					
Acute kidney injury	15 248 (20.2)	12 074 (19.8)	3079 (21.7)	1340 (18.5)	4073 (20.5)
Anaphylaxis	12 (0.01)	10 (0.02)	2 (0.01)	1 (0.01)	3 (0.01)
Bacteremia	319 (0.4)	240 (0.4)	76 (0.6)	26 (0.4)	78 (0.4)
Rhabdomyolysis	1125 (1.5)	968 (1.6)	150 (1.0)	96 (1.3)	329 (1.6)
Sepsis	17 569 (23.3)	14 073 (23.1)	3378 (24.1)	1963 (27.2)	4557 (22.8)
Transplant	467 (0.6)	348 (0.5)	116 (0.8)	62 (0.8)	129 (0.6)

Abbreviation: FluSurv-NET, US Influenza Hospitalization Surveillance Network.

^a^ Patients for whom both influenza A and B (n = 310) were detected and for whom influenza A and B could not be distinguished (n = 174) were included in the total but not further included in this table. Influenza A subtyping was performed for a subset of patients with influenza A infection (n = 27 449). Patients for whom both influenza A(H1N1)pdm09 and influenza A(H3N2) were detected were not included in subtype data (n = 12). Numbers are unweighted values, and percentages are weighted values. Percentages are column percentages.

Patients with pneumonia, sepsis, and acute kidney injury, which were the most common diagnoses overall, had a high frequency of severe hospital outcomes, including ICU admission (26%-33%) and in-hospital mortality (6%-8%) (Table 3 and eTable 4 in the Supplement). Other severe acute diagnoses were rare, including acute gastrointestinal tract and neurologic diagnoses, but were associated with a disproportionately high frequency of ICU admission and in-hospital mortality. Among patients with acute gastrointestinal tract diagnoses (of whom half had acute hepatitis or hepatic failure), 45.4% were admitted to the ICU, 33.0% received mechanical ventilatory assistance, and 18.9% died in the hospital. Among patients with acute neurologic diagnoses (most commonly cerebral ischemia), 35.3% required ICU admission and 12.0% died in the hospital.

**Table 3.** In-Hospital Outcomes Among Adults Hospitalized With Laboratory-Confirmed Influenza by Acute Diagnosis Category in FluSurv-NET, United States, 2010-2018^a^

Acute diagnosis	Total, No. (%)	Length of stay, median (IQR), d	Patients, No. (%)			
			Intensive care unit admission	Mechanical ventilatory support	Extracorporeal membrane oxygenation	In-hospital mortality
Any	76 649 (100)	3 (2-5)	12 549 (16.0)	5163 (6.5)	223 (0.3)	2644 (3.2)
Respiratory tract	72 997 (94.9)	3 (2-5)	11 843 (15.9)	4970 (6.6)	209 (0.3)	2488 (3.1)
Sepsis	17 569 (23.3)	4 (2-7)	5959 (32.6)	3000 (16.1)	102 (0.6)	1632 (8.3)
Acute kidney injury	15 248 (20.2)	4 (2-7)	4483 (28.4)	2200 (13.7)	86 (0.6)	1263 (7.4)
Cardiovascular	9046 (12.1)	5 (3-8)	2901 (31.2)	1319 (14.0)	40 (0.4)	740 (7.3)
Hematologic	1488 (1.9)	5 (3-10)	510 (34.3)	297 (19.8)	18 (1.2)	165 (10.5)
Rhabdomyolysis	1125 (1.5)	4 (3-7)	217 (18.3)	93 (7.7)	7 (0.6)	49 (4.0)
Endocrine	1143 (1.4)	3 (2-5)	552 (47.8)	122 (10.5)	3 (0.3)	41 (3.4)
Gastrointestinal tract	1038 (1.3)	4 (2-10)	495 (45.4)	370 (33.0)	22 (2.0)	213 (18.9)
Neurologic	939 (1.2)	5 (3-10)	343 (35.3)	209 (21.2)	6 (0.6)	123 (12.0)

Abbreviations: FluSurv-NET, US Influenza Hospitalization Surveillance Network; IQR, interquartile range.

^a^ Numbers are unweighted values, and percentages are weighted values. Acute diagnosis categories are not mutually exclusive unless otherwise stated.

## Discussion

Using a large population-based surveillance system, we examined the spectrum of acute diagnoses in more than 80 000 adults hospitalized with laboratory-confirmed influenza across 8 influenza seasons. Although most patients received acute respiratory diagnoses, including pneumonia and acute respiratory failure, almost half received acute nonrespiratory diagnoses; the most common nonrespiratory diagnoses included sepsis and acute kidney injury. In many patients, severe outcomes and the demand for hospital resources were greater for patients with nonrespiratory diagnoses. The results suggest that a full appreciation of the spectrum of disease and the burden associated with influenza should consider the potential respiratory and nonrespiratory diagnoses.

The frequency of diagnoses in this analysis was similar to findings from studies^3,17^ conducted before the 2009 pandemic. One such analysis^17^ included data from FluSurv-NET from 2005 to 2009. Although differences were found in how certain diagnoses were categorized in that study^17^ compared with ours, the frequency of seasonal influenza-associated pneumonia and chronic obstructive pulmonary disease exacerbation was similar. For certain diagnoses, such as acute kidney injury, the frequency of diagnoses was higher in the present study compared with studies conducted before 2009. These differences may be the result of changes in *ICD-9-CM* and *ICD-10* coding over time, increased influenza testing after the 2009 influenza pandemic, or changes in influenza viruses in circulation.

Across 8 influenza seasons, patients with acute nonrespiratory diagnoses only were found to be less likely to receive antiviral treatment compared with those with respiratory diagnoses, suggesting possible missed opportunities to most effectively manage influenza infections in hospitalized patients. In the United States, influenza antiviral treatment is recommended for any patient who is hospitalized with suspected or confirmed influenza ^21^ because observational studies^22,23^ have found a survival benefit associated with antiviral treatment, especially in situations in which treatment was initiated early after onset of symptoms. Sepsis was the most common nonrespiratory diagnosis in the present analysis and presents another potential opportunity for effective management of influenza-associated diagnoses. Sepsis is often attributed to bacterial infections, but influenza is also capable of triggering the same physiologic sepsis cascade as the direct result of the viral infection or by secondary bacterial infection.^13^ During the influenza season, early suspicion of influenza in patients with sepsis without another identifiable cause may help reduce the need for antibiotic treatment^24^ and ensure earlier implementation of antiviral treatment.^25,26^

These findings suggest that a broad range of diagnoses may be associated with influenza in hospitalized patients and highlight the severe in-hospital outcomes that may be associated with influenza virus infection in adult patients. Pneumonia, sepsis, and acute kidney injury were associated with in-hospital mortality of 6% to 8%. These diagnoses comprised 20% to 36% of all influenza-associated diagnoses. Patients with certain less common diagnoses had among the highest frequencies of severe outcomes. In particular, acute gastrointestinal tract diagnoses were associated with the highest frequency of mechanical ventilatory assistance and in-hospital mortality among the nonrespiratory diagnoses. These frequencies were primarily associated with diagnosis of acute hepatitis or acute hepatic failure. Previous studies^27,28,29^ have explored the hepatic complications of influenza infection. Marzano et al^27^ described a cluster of hospital-acquired influenza A (H1N1)pdm09 infections among patients admitted to a hepatology unit, and despite antiviral treatment, 3 of the 4 patients with underlying cirrhosis died of multiorgan failure. Other studies^28,29^ have suggested that influenza may also be associated with hepatic inflammation. A potential mechanism for secondary hepatitis associated with influenza virus infection was proposed by Polakos et al^28^ in which a constellation of immune cell reactivity that involved hepatocytes, CD8^+^ T cells, and Kupffer cells led to histologic and serum evidence of hepatitis. Literature on the topic is sparse to date, and further exploration of the acute gastrointestinal tract diagnoses associated with influenza virus infection should be pursued.

Among this large population of adults hospitalized with laboratory-confirmed influenza, only 82% received an influenza-specific *ICD* code. We further found that using pneumonia or influenza *ICD* codes failed to capture 14% of patients with laboratory-confirmed influenza reported to FluSurv-NET. These findings have implications for the potential underestimation of rates in studies that identify influenza using administrative data. Such administratively based studies could increase the sensitivity of their case definition by expanding to other respiratory and circulatory codes or selective groups of nonrespiratory codes.

### Limitations

This study has limitations. We identified acute diagnoses by *ICD* codes, a method commonly used to classify patient diagnoses in several studies of influenza complications,^8,30^ which may have inherent limitations that led to misclassification.^31^ For example, there may be differences in how medical coders assign *ICD* codes or in how codes are assigned for billing purposes or insurance reimbursement. There may be further misclassification in some instances because *ICD* codes could not distinguish between acute diagnoses that occur during hospitalization and chronic underlying conditions or diagnoses made before admission. To help reduce misclassification, we chose to limit *ICD* codes to those most likely to be associated with an acute process. Furthermore, some diagnoses may have been reported concurrently during a patient’s hospitalization and may not have been directly associated with influenza virus infection. A further limitation is that data are reported to FluSurv-NET based on practitioner-ordered influenza diagnostic tests. Because testing practices may vary by hospital site, presenting symptoms, or the type of acute diagnosis present at admission, the estimates of the frequency of nonrespiratory diagnoses is likely underestimated. In some cases, testing may depend on symptoms associated with influenza. Not all patients present with symptoms that fulfill the criteria for influenza-like illness, with some study findings^32,33^ suggesting that only 50% to 79% of adults with influenza will meet the influenza-like illness definition. In situations when a patient does not present with recognizable influenza symptoms, such as influenza-like illness, practitioners may not test for influenza. In addition, FluSurv-NET only collects the first 9 *ICD* codes listed in the patient’s medical record; thus, diagnoses listed after the first 9 were not analyzed.

## Conclusions

In this study, hospitalized patients with influenza commonly had acute respiratory diagnoses, such as pneumonia, but also had a variety of nonrespiratory diagnoses. The diagnoses associated with seasonal influenza highlight the burden of influenza on the population and the health care system and suggest the range of influenza-related diagnoses that should be considered by health care practitioners when patients are hospitalized with influenza virus infection.

## Notes

Supplement. **eTable 1.** *International Classification of Diseases* Codes (*ICD-9* and *ICD-10*) by Acute Diagnosis **eTable 2.** Most Common *International Classification of Diseases* Codes in Patients not Categorized into an Acute Diagnosis Group, FluSurv-NET, United States, 2010-2018 **eTable 3.** Frequency of Acute Respiratory and Nonrespiratory Diagnoses in Hospitalized Adults with Influenza by Influenza (Sub)type, FluSurv-NET, United States, 2010-2018 **eTable 4.** In-Hospital Outcomes by Specific Acute Respiratory Diagnoses, FluSurv-NET, United States, 2010-2018 **eFigure 1.** Venn Diagram of Acute Respiratory and Nonrespiratory Diagnoses, FluSurv-NET, United States, 2010-2018 **eFigure 2.** *ICD* Code–Based Identification of Influenza and Related Diagnoses Among Adult Patients Hospitalized with Influenza, FluSurv-NET, United States, 2010-2018
