# Supplementary material for: Respiratory and Nonrespiratory Diagnoses Associated With Influenza in Hospitalized Adults
Source: JAMA Netw Open. 2020 Mar 20;3(3):e201323. doi: 10.1001/jamanetworkopen.2020.1323 (PMC7084169; doi:10.1001/jamanetworkopen.2020.1323)
Supplement: Supplement. — eTable 1. International Classification of Diseases Codes (ICD-9 and ICD-10) by Acute Diagnosis eTable 2. Most Common International Classification of Diseases Codes in Patients not Categorized into an Acute Diagnosis Group, FluSurv-NET, United States, 2010-2018 eTable 3. Frequency of Acute Respiratory and Nonrespiratory Diagnoses in Hospitalized Adults with Influenza by Influenza (Sub)type, FluSurv-NET, United States, 2010-2018 eTable 4. In-Hospital Outcomes by Specific Acute Respiratory Diagnoses, FluSurv-NET, United States, 2010-2018 eFigure 1. Venn Diagram of Acute Respiratory and Nonrespiratory Diagnoses, FluSurv-NET, United States, 2010-2018 eFigure 2. ICD Code–Based Identification of Influenza and Related Diagnoses Among Adult Patients Hospitalized with Influenza, FluSurv-NET, United States, 2010-2018 [file jamanetwopen-3-e201323-s001.pdf]

Supplementary Online Content

Chow EJ, Rolfes MA, O'Halloran A, et al. Respiratory and nonrespiratory diagnoses associated with influenza in hospitalized adults. *JAMA Netw Open*. 2020;3(3):e201323. doi:10.1001/jamanetworkopen.2020.1323

eTable 1. *International Classification of Diseases* Codes (*ICD-9* and *ICD-10*) by Acute Diagnosis

eTable 2. Most Common *International Classification of Diseases* Codes in Patients not Categorized into an Acute Diagnosis Group, FluSurv-NET, United States, 2010-2018

eTable 3. Frequency of Acute Respiratory and Nonrespiratory Diagnoses in Hospitalized Adults with Influenza by Influenza (Sub)type, FluSurv-NET, United States, 2010-2018

eTable 4. In-Hospital Outcomes by Specific Acute Respiratory Diagnoses, FluSurv-NET, United States, 2010-2018

eFigure 1. Venn Diagram of Acute Respiratory and Nonrespiratory Diagnoses, FluSurv-NET, United States, 2010-2018

eFigure 2. *ICD* Code–Based Identification of Influenza and Related Diagnoses Among Adult Patients Hospitalized with Influenza, FluSurv-NET, United States, 2010-2018

This supplementary material has been provided by the authors to give readers additional information about their work.

© 2020 Chow EJ et al. *JAMA Network Open.*

eTable 1. *International Classification of Diseases* Codes (*ICD-9* and *ICD-10*) by Acute Diagnosis

DISEASE CONDITION	*ICD-9*	*ICD-10*
**ACUTE RESPIRATORY TRACT DIAGNOSES**		
**Acute Respiratory Distress Syndrome**		
Acute respiratory distress syndrome	518.82	J80
**Acute Upper Respiratory Infections**		
Acute upper respiratory infections	460-465	J00-J06
**Asthma Exacerbation**		
Mild intermittent asthma with acute exacerbation	-	J45.21
Mild intermittent asthma with status asthmaticus	-	J45.22
Mild persistent asthma with acute exacerbation	-	J45.31
Mild persistent asthma with status asthmaticus	-	J45.32
Moderate persistent asthma with acute exacerbation	-	J45.41
Moderate persistent asthma with status asthmaticus	-	J45.42
Severe persistent asthma with acute exacerbation	-	J45.51
Severe persistent asthma with status asthmaticus	-	J45.52
Unspecified asthma with acute exacerbation	493.92	J45.901
Unspecified asthma with status asthmaticus	493.91	J45.902
Extrinsic asthma with status asthmaticus	493.01	-
Extrinsic asthma with acute exacerbation	493.02	-
Intrinsic asthma with status asthmaticus	493.11	-
Intrinsic asthma with acute exacerbation	493.12	-
Chronic obstructive asthma with status asthmaticus	493.21	-
Chronic obstructive asthma with acute exacerbation	493.22	-
**Chronic Obstructive Pulmonary Disease Exacerbation**		
COPD with acute exacerbation	491.21	J44.1
**Mediastinitis**		
Mediastinitis	519.2	J98.51
**Other Acute Lower Respiratory Tract Disease**		

© 2020 Chow EJ et al. *JAMA Network Open.*

Acute bronchitis	466.0	J20
Acute bronchiolitis	466.1	J21
Unspecified acute lower respiratory infection	519.8	J22
COPD with acute lower respiratory infection	491.22	J44.0
Bronchiectasis with acute lower respiratory infection	-	J47.1
Bronchiectasis with acute exacerbation	494.1	J47.9
Abscess of lung and mediastinum	513	
Gangrene and necrosis of lung	-	J85.0
Abscess of lung without pneumonia	513.0	J85.2
Abscess of mediastinum	513.1	J85.3
Pyothorax	510	J86
Pyothorax with fistula	510.0	J86.0
Pyothorax without fistula	510.9	J86.9
**Pneumonia**		
Viral pneumonia	480	J12
Streptococcus pneumoniae pneumonia	481	J13
Hemophilus influenzae pneumonia	482.2	J14
Other bacterial pneumonia	482	J15
Pneumonia due to other specified organism	483	J16
Pneumonia in infectious diseases classified elsewhere	484	J17
Pneumonia, unspecified organism	486	J18
Bronchopneumonia, organism unspecified	485	-
Abscess of lung with pneumonia	513.0	J85.1
Influenza due to identified novel influenza A virus with pneumonia	488.81	J09.X1
Influenza due to other identified influenza virus with pneumonia	488.01	J10.0
Influenza due to unidentified influenza virus with pneumonia	-	J11.0
Influenza due to identified 2009 H1N1 influenza virus with pneumonia	488.11	-
Influenza with pneumonia	487.0	-
**Pneumothorax**		
Pneumothorax and air leak	512	J93

© 2020 Chow EJ et al. *JAMA Network Open.*

**Pulmonary Collapse**		
Atelectasis	518.0	J98.11
Other pulmonary collapse	518.0	J98.19
**Respiratory Failure**		
Acute respiratory failure	518.81	J96.0
Acute and chronic respiratory failure	518.84	J96.2
Respiratory arrest	799.1	R09.2
**Influenza with Other Respiratory Manifestations**		
Influenza due to identified novel influenza A virus with other respiratory manifestations	-	J09.X2
Influenza due to other identified influenza virus with other respiratory manifestations	-	J10.1
Influenza due to unidentified influenza virus with other respiratory manifestations	-	J11.1
Influenza with other respiratory manifestations	487.1	-
Influenza due to identified avian influenza with other respiratory manifestations	488.02	-
Influenza due to identified 2009 H1N1 influenza virus with other respiratory manifestations	488.12	-
Influenza due to identified novel influenza A virus with other respiratory manifestations	488.82	-
**INFLUENZA WITH OTHER MANIFESTATIONS**		
**Influenza with Other Manifestations**		
Influenza with other manifestations	487.8	-
Influenza due to identified avian influenza virus with other manifestations	488.09	-
influenza due to identified 2009 H1N1 influenza virus with other manifestations	488.19	-
Influenza due to identified novel influenza A virus with other manifestations	488.89	-
Influenza due to identified novel influenza A virus with other manifestations	-	J09.X9
Influenza due to other identified influenza virus with otitis media	-	J10.83
Influenza due to other identified influenza virus with other manifestations	-	J10.89
Influenza due to unidentified influenza virus with otitis media	-	J11.83
Influenza due to unidentified influenza virus with other manifestations	-	J11.89
Influenza due to other identified influenza virus with encephalopathy	-	J10.81
Influenza due to unidentified influenza virus with encephalopathy	-	J11.81
**ACUTE NEUROLOGIC DIAGNOSES**		
**Acute Disseminated Encephalitis and Encephalomyelitis**		

© 2020 Chow EJ et al. *JAMA Network Open.*

Acute disseminated encephalitis and encephalomyelitis (ADEM)	323.61	G04.0
**Cerebral Ischemia/Infarction**		
Cerebral Infarction	434	I63
Acute cerebrovascular insufficiency	437.1	I67.81
Cerebral ischemia	437.1	I67.82
Transient cerebral ischemic attacks and related syndromes	435	G45
Transient global amnesia	437.7	G45.4
**Demyelinating Disease**		
Other acute disseminated demyelination	341.8	G36
**Encephalitis**		
Encephalitis, myelitis and encephalomyelitis	323	G04
Encephalitis, myelitis and encephalomyelitis in diseases classified elsewhere	323.4	G05
**Guillain-Barre Syndrome**		
Guillain-Barre syndrome	357.0	G61.0
**Intracranial Hemorrhage**		
Nontraumatic subarachnoid hemorrhage	430	I60
Nontraumatic intracerebral hemorrhage	431	I61
Other and unspecified nontraumatic intracranial hemorrhage	432	I62
**Meningitis**		
Bacterial meningitis, not elsewhere classified	320	G00
Meningitis in bacterial diseases classified elsewhere	320	G01
Meningitis in other infectious and parasitic diseases classified elsewhere	321	G02
Meningitis due to other and unspecified causes	322	G03
**ACUTE CARDIOVASCULAR EVENTS**		
**Acute Myocarditis**		
Acute myocarditis	422	I40
Influenza due to other identified influenza virus with myocarditis	-	J10.82
Influenza due to unidentified influenza virus with myocarditis	-	J11.82
**Acute Pericarditis**		
Acute pericarditis	420	I30

© 2020 Chow EJ et al. *JAMA Network Open.*

**Cardiac Tamponade**		
Cardiac tamponade	423.3	I31.4
**Cardiogenic Shock**		
Cardiogenic shock	785.51	R57.0
**Congestive Heart Failure**		
Acute systolic heart failure	428.21	I50.21
Acute on chronic systolic heart failure	428.23	I50.23
Acute diastolic heart failure	428.31	I50.31
Acute on chronic diastolic heart failure	428.33	I50.33
Acute combined systolic and diastolic heart failure	428.41	I50.41
Acute on chronic combined systolic and diastolic heart failure	428.43	I50.43
Acute right heart failure	428.9	I50.811
Acute on chronic right heart failure	428.9	I50.813
**Hypertensive Crisis**	-	I16
Malignant essential hypertension	401.0	-
Malignant hypertensive heart disease	402.0	-
Malignant hypertensive heart disease without heart failure	402.00	-
Malignant hypertensive heart disease with heart failure	402.01	-
Malignant hypertensive renal disease	403.0	-
Malignant hypertensive heart and renal disease	404.0	-
Malignant secondary hypertension	405.0	-
Malignant renovascular hypertension	405.01	-
Other malignant secondary hypertension	405.09	-
Hypertensive urgency	-	I16.0
Hypertensive emergency	-	I16.1
Hypertensive crisis, unspecified	-	I16.9
**Ischemic Heart Disease**		
Unstable angina	411.1	I20.0
Acute myocardial infarction	410	I21
Acute myocardial infarction of anterolateral wall	410.0	-

© 2020 Chow EJ et al. *JAMA Network Open.*

Acute myocardial infarction of other anterior wall	410.1	-
Acute myocardial infarction of inferolateral wall	410.2	-
Acute myocardial infarction of inferoposterior wall	410.3	-
Acute myocardial infarction of other inferior wall	410.4	-
Acute myocardial infarction of other lateral wall	410.5	-
True posterior wall infarction	410.6	-
Subendocardial infarction	410.7	-
Acute myocardial infarction of other specified sites	410.8	-
Acute myocardial infarction of unspecified site	410.9	-
ST elevation myocardial infarction of anterior wall	-	I21.0
ST elevation myocardial infarction of inferior wall	-	I21.1
ST elevation myocardial infarction of other sites	-	I21.2
ST elevation myocardial infarction of unspecified site	-	I21.3
Non-ST elevation myocardial infarction	410.71	I21.4
Acute myocardial infarction, unspecified	-	I21.9
Subsequent ST elevation and non-ST elevation myocardial infarction	410.01-410.11	I22
Subsequent ST elevation myocardial infarction of anterior wall	410.21, 410.31, 410.41	I22.0
Subsequent ST elevation myocardial infarction of inferior wall	410.21, 410.31, 410.41	I22.1
Subsequent non-ST elevation myocardial infarction	410.71	I22.2
Subsequent ST elevation myocardial infarction of other sites	410.51, 410.61, 410.81	I22.8
Subsequent ST elevation myocardial infarction of unspecified site	410.91	I22.9
Other acute and subacute forms of ischemic heart disease	411	I24
**ACUTE ENDOCRINE DIAGNOSES**		
**Diabetic Ketoacidosis**		
Diabetes mellitus due to underlying condition with ketoacidosis	-	E08.1
…without coma	-	E08.10
…with coma	-	E08.11

© 2020 Chow EJ et al. *JAMA Network Open.*

Drug or chemical induced diabetes mellitus with ketoacidosis	-	E09.1
…without coma	-	E09.10
…with coma	-	E09.11
Type 1 diabetes mellitus with ketoacidosis	-	E10.1
…without coma	-	E10.10
…with coma	-	E10.11
Type 2 diabetes mellitus with ketoacidosis	-	E11.1
…without coma	-	E11.10
…with coma	-	E11.11
Other specified diabetes mellitus with ketoacidosis	-	E13.1
…without coma	-	E13.10
…with coma	-	E13.11
Secondary diabetes mellitus with ketoacidosis	249.1	-
Secondary diabetes mellitus with ketoacidosis, not stated as uncontrolled, or unspecified	249.10	-
Secondary diabetes mellitus with ketoacidosis, uncontrolled	249.11	-
Diabetes with ketoacidosis	250.1	-
Diabetes with ketoacidosis, type II or unspecified type, not stated as uncontrolled	250.10	-
Diabetes with ketoacidosis, type I, not stated as uncontrolled	250.11	-
Diabetes with ketoacidosis, type II or unspecified type, uncontrolled	250.12	-
Diabetes with ketoacidosis, type I, uncontrolled	250.13	-
**Hyperglycemic Hyperosmolar Syndrome**		
Diabetes mellitus due to underlying condition with hyperosmolarity	-	E08.0
…without nonketotic hyperglycemic-hyperosmolar coma	-	E08.00
…with coma	-	E08.01
Drug or chemical induced diabetes mellitus with hyperosmolarity	-	E09.0
...without nonketotic hyperglycemic-hyperosmolar coma	-	E09.00
…with coma	-	E09.01
Type 2 diabetes mellitus with hyperosmolarity	-	E11.0
…without nonketotic hyperglycemic-hyperosmolar coma	-	E11.00
…with coma	-	E11.01

© 2020 Chow EJ et al. *JAMA Network Open.*

Other specified diabetes mellitus with hyperosmolarity	-	E13.0
…without nonketotic hyperglycemic-hyperosmolar coma	-	E13.00
…with coma	-	E13.01
Secondary diabetes mellitus with hyperosmolarity	249.2	-
Secondary diabetes mellitus with hyperosmolarity, not stated as uncontrolled, or unspecified	249.20	-
Secondary diabetes mellitus with hyperosmolarity with hyperosmolarity, uncontrolled	249.21	-
Diabetes with hyperosmolarity	250.2	-
Diabetes with hyperosmolarity, type II or unspecified type, not stated as uncontrolled	250.20	-
Diabetes with hyperosmolarity, type I, not stated as uncontrolled	250.21	-
Diabetes with hyperosmolarity, type II or unspecified type, uncontrolled	250.22	-
Diabetes with hyperosmolarity, type I, uncontrolled	250.23	-
**Thyrotoxicosis**		
Thyrotoxicosis	242	E05
**ACUTE GASTROINTESTINAL TRACT DIAGNOSES**		
**Acute Hepatitis and Hepatic Failure**		
Acute and subacute hepatic failure	570	K72.0
Acute viral hepatitis	070.9	-
Viral hepatitis A with hepatic coma	070.0	-
Viral hepatitis B with hepatic coma	070.2	-
Other specified viral hepatitis with hepatic coma	070.4	-
Unspecified viral hepatitis with hepatic coma	070.6	-
Unspecified viral hepatitis C with hepatic coma	070.71	-
Acute hepatitis A	-	B15
Acute hepatitis B	-	B16
Other acute viral hepatitis	-	B17
Unspecified viral hepatitis with hepatic coma	-	B19.0
Unspecified viral hepatitis B with hepatic coma	-	B19.11
Unspecified viral hepatitis C with hepatic coma	-	B19.21
**Acute Pancreatitis**		
Acute pancreatitis	577.0	K85

© 2020 Chow EJ et al. *JAMA Network Open.*

**Influenza with Gastrointestinal Manifestations**		
Influenza due to identified novel influenza A virus with gastrointestinal manifestations	-	J09.X3
Influenza due to other identified influenza virus with gastrointestinal manifestations	-	J10.2
Influenza due to unidentified influenza virus with gastrointestinal manifestations	-	J11.2
**ACUTE HEMATOLOGIC DIAGNOSES**		
**Disseminated Intravascular Coagulation**		
Disseminated intravascular coagulation	286.6	D65
**Deep Vein Thrombosis**		
Acute embolism and thrombosis of superior vena cava	-	I82.210
Acute embolism and thrombosis of other thoracic veins	-	I82.290
Acute embolism and thrombosis of inferior vena cava	-	I82.220
Acute embolism and thrombosis of unspecified deep veins of lower extremity	-	I82.4
Acute embolism and thrombosis of veins of upper extremity	-	I82.6
Acute embolism and thrombosis of axillary vein	-	I82.A1
Acute embolism and thrombosis of subclavian vein	-	I82.B1
Acute embolism and thrombosis of internal jugular vein	-	I82.C1
Acute venous embolism and thrombosis of deep vessels of lower extremity	453.4	-
Acute venous embolism and thrombosis of deep veins of upper extremity	453.82	-
Acute venous embolism and thrombosis of axillary veins	453.84	-
Acute venous embolism and thrombosis of subclavian veins	453.85	-
Acute venous embolism and thrombosis of internal jugular veins	453.86	-
Acute venous embolism and thrombosis of other thoracic veins	453.87	-
**Hemophagocytic Syndrome**		
Hemophagocytic lymphohistiocytosis	-	D76.1
Hemophagocytic syndromes	288.4	-
Hemophagocytic syndrome, infection-associated	-	D76.2
**Immune Thrombocytopenic Purpura**		
Immune thrombocytopenic purpura	287.31	D69.3
**Pulmonary Embolism**		
Pulmonary embolism and infarction	415.1	-

© 2020 Chow EJ et al. *JAMA Network Open.*

Pulmonary embolism	-	I26
**Sickle Cell Crisis**		
Hb-SS disease with crisis	282.62	D57.0
Hb-SS with acute chest syndrome	517.3	D57.01
Hb-SS disease with splenic sequestration	289.52	D57.02
Sickle-cell/Hb-C disease with crisis	282.64	D57.21
Sickle-cell/Hb-C disease with acute chest syndrome	517.3	D57.211
Sickle-cell/Hb-C disease with splenic sequestration	289.52	D57.212
Sickle-cell thalassemia with crisis	282.42	D57.41
Sickle-cell thalassemia with acute chest syndrome	517.3	D57.411
Sickle-cell thalassemia with splenic sequestration	289.52	D57.412
Other sickle-cell disorders with crisis	282.69	D57.81
Other sickle-cell disorders with acute chest syndrome	517.3	D57.811
Other sickle-cell disorders with splenic sequestration	289.52	D57.812
**OTHER ACUTE DIAGNOSES**		
**Acute Kidney Injury**		
Acute kidney failure	584	N17
**Anaphylaxis**		
Other anaphylactic reaction	995.0	-
Anaphylactic shock, unspecified, initial encounter	-	T782XXA
**Bacteremia**	790.7	R78.81
**Rhabdomyolysis**		
Rhabdomyolysis	728.88	M62.82
**Sepsis**		
Septicemia	038	-
Systemic inflammatory response syndrome	995.9	-
Streptococcal sepsis	-	A40
Other sepsis	-	A41
Symptoms and signs specifically associated with systemic inflammation and infection	-	R65
**Transplant**		

© 2020 Chow EJ et al. *JAMA Network Open.*

Complications of transplanted organ	996.8	T86
**All Influenza Codes (Summarized)**		
**Influenza**	487	-
**Influenza due to certain identified influenza viruses**	488	J09
**Influenza due to other identified influenza virus**	-	J10
**Influenza due to unidentified influenza virus**	-	J11

© 2020 Chow EJ et al. *JAMA Network Open.*

eTable 2. Most Common *International Classification of Diseases* Codes in Patients not Categorized into an Acute Diagnosis Group, FluSurv-NET, United States, 2010-2018

ICD	Code	*ICD* Code Name
9	401.9	Unspecified essential hypertension
9	272.4	Other and unspecified hyperlipidemia
9	250.00	Diabetes mellitus without mention of complication
9	276.51	Dehydration
9	427.31	Atrial fibrillation
9	780.60	Fever, unspecified
9	530.81	Esophageal reflux
9	276.8	Hypopotassemia
9	487.8	Influenza with other manifestations
9	786.2	Cough
10	I10	Essential (primary) hypertension
10	E78.5	Hyperlipidemia, unspecified
10	E11.9	Type 2 diabetes mellitus without complications
10	E86.0	Dehydration
10	I25.10	Chronic ischemic heart disease without angina pectoris
10	E03.9	Hypothyroidism, unspecified
10	K21.9	Gastro-esophageal reflux disease without esophagitis
10	E87.1	Hypo-osmolality and hyponatremia
10	F32.9	Major depressive disorder, single episode, unspecified
10	J10.89	Influenza due to other identified influenza virus with other manifestations

© 2020 Chow EJ et al. *JAMA Network Open.*

eTable 3. Frequency of Acute Respiratory and Nonrespiratory Diagnoses in Hospitalized Adults with Influenza by Influenza (Sub)type, FluSurv-NET, United States, 2010-2018[a]

**Acute Diagnoses**	**Total**		**Influenza A**		**Influenza B**		**Influenza A (H1N1)**		**Influenza A (H3N2)**	
	N= 76649		N = 61862		N= 14303		N=7243		N = 20194	
	N	% of Total Observations	N	% of Influenza A	N	% of Influenza B	N	% of Influenza A (H1N1)	N	% of Influenza A (H3N2)
**Respiratory Tract Diagnoses**	72997	94.9	59052	95.2	13489	93.7	6974	96.3	19214	95.0
Acute Respiratory Distress Syndrome	581	0.7	480	0.7	94	0.6	101	1.4	120	0.6
Acute Upper Respiratory Infection	1393	1.7	1107	1.7	278	1.8	142	1.9	438	2.1
Asthma Exacerbation	7107	8.9	5908	9.2	1165	7.7	836	11.4	1841	8.8
Chronic Obstructive Pulmonary Disease Exacerbation	11081	14.7	8965	14.7	2061	14.7	1196	16.8	3059	15.4
Mediastinitis	12	0.01	4	0.006	8	0.05	1	0.01	0	0
Other Acute Lower Respiratory Tract Disease	6473	8.8	5019	8.4	1415	10.5	548	7.8	1844	9.2
Pneumonia	28026	36.3	22583	36.2	5233	36.4	3163	43.5	6923	34.0
Pneumothorax	167	0.2	125	0.2	41	0.3	25	0.3	23	0.1
Pulmonary Collapse	1269	1.6	1014	1.6	246	1.6	137	1.9	322	1.6
Respiratory Failure	20143	26.6	16258	26.5	3760	26.6	2232	31.2	5607	28.2
Influenza with Other Respiratory Manifestations	43125	56.1	35056	56.6	7844	54.4	3583	49.6	11458	56.8
**Influenza with Other Manifestations**	781	1.0	640	1.1	137	1.0	83	1.1	193	0.9
**Neurologic Diagnosis**	939	1.2	739	1.2	192	1.3	85	1.2	241	1.2
Acute Disseminated Encephalitis and Encephalomyelitis (ADEM)	4	0.006	2	0.003	2	0.02	1	0.01	0	0
Cerebral Ischemia or Infarction	657	0.9	519	0.8	130	0.9	55	0.8	172	0.8
Demyelinating Disease	7	0.008	5	0.008	2	0.01	2	0.03	2	0.009
Encephalitis	45	0.06	39	0.06	6	0.05	7	0.09	10	0.05

© 2020 Chow EJ et al. *JAMA Network Open.*

Guillain-Barre Syndrome	41	0.05	37	0.06	4	0.02	4	0.05	13	0.07
Intracranial Hemorrhage	157	0.2	116	0.2	40	0.2	11	0.2	38	0.2
Meningitis	65	0.08	49	0.1	16	0.1	8	0.1	14	0.06
**Cardiovascular Event**	9046	12.1	7319	12.1	1671	12.0	690	9.6	2642	13.4
Acute Myocarditis	74	0.1	52	0.08	22	0.2	5	0.07	19	0.09
Acute Pericarditis	42	0.06	30	0.05	11	0.08	3	0.04	7	0.03
Cardiac Tamponade	19	0.03	17	0.03	2	0.01	2	0.03	6	0.03
Cardiogenic Shock	261	0.3	207	0.3	52	0.3	39	0.5	65	0.3
Congestive Heart Failure	4828	6.5	3889	6.5	908	6.4	373	5.3	1407	7.1
Hypertensive Crisis	788	1.0	619	1.0	162	1.1	51	0.8	204	1.0
Ischemic Heart Disease	4412	6.0	3614	6.0	773	5.7	325	4.6	1347	6.9
**Endocrine Diagnosis**	1143	1.4	883	1.4	251	1.6	121	1.6	280	1.4
Diabetic Ketoacidosis	822	1.0	616	1.0	199	1.3	91	1.2	192	0.9
Hyperglycemic Hyperosmolar Syndrome	74	0.1	57	0.1	16	0.1	6	0.08	15	0.07
Thyrotoxicosis	251	0.3	214	0.3	36	0.2	25	0.3	73	0.4
**Gastrointestinal Tract Diagnosis**	1038	1.3	792	1.3	229	1.6	140	1.9	248	1.2
Acute Hepatitis or Liver Failure	524	0.7	414	0.6	103	0.7	74	1.0	137	0.7
Acute Pancreatitis	214	0.3	161	0.3	47	0.3	31	0.4	44	0.2
Influenza with Gastrointestinal Manifestations	311	0.4	227	0.4	80	0.6	36	0.5	70	0.4
**Hematologic Diagnosis**	1488	1.9	1196	1.9	279	1.8	178	2.4	357	1.8
Disseminated Intravascular Coagulation	131	0.2	98	0.2	31	0.2	25	0.3	23	0.1
Deep Vein Thrombosis	482	0.6	398	0.6	79	0.5	62	0.9	115	0.6
Hemophagocytic Syndrome	5	0.006	4	0.006	1	0.006	0	0	4	0.02
Immune Thrombocytopenic Purpura	177	0.2	157	0.3	19	0.1	15	0.2	63	0.3
Pulmonary Embolism	569	0.7	457	0.7	103	0.7	66	0.9	133	0.7
Sickle Cell Crisis	218	0.3	157	0.2	61	0.4	23	0.3	40	0.2

© 2020 Chow EJ et al. *JAMA Network Open.*

**Other Diagnoses**											
Acute Kidney Injury	15248	20.2	12074	19.8	3079	21.7		1340	18.5	4073	20.5
Anaphylaxis	12	0.01	10	0.02	2	0.01		1	0.01	3	0.01
Bacteremia	319	0.4	240	0.4	76	0.6		26	0.4	78	0.4
Rhabdomyolysis	1125	1.5	968	1.6	150	1.0		96	1.3	329	1.6
Sepsis	17569	23.3	14073	23.1	3378	24.1		1963	27.2	4557	22.8
Transplant Complications	467	0.6	348	0.5	116	0.8		62	0.8	129	0.6

[a]Numbers are unweighted values, and percentages are weighted values. Percentages are column percentages. Patients for whom both influenza A and B were detected (n = 310) and for whom influenza A and B could not be distinguished (n= 174) were included in the total but not further included in this table. Influenza A subtyping was performed for a subset of patients with influenza A infection (n = 27449). Patients for whom both influenza A(H1N1)pdm09 and influenza A(H3N2) were detected were not included in subtype data (n = 12).

© 2020 Chow EJ et al. *JAMA Network Open.*

eTable 4. In-Hospital Outcomes by Specific Acute Respiratory Diagnoses, FluSurv-NET, United States, 2010-2018[a]

	Total		Length of Stay (Days)		Intensive Care Unit Admission		Mechanical Ventilatory Support		Extra Corporeal Membrane Oxygenation		In-Hospital Mortality	
	N	%	Median	Interquartile Range	N	%	N	%	N	%	N	%
**Any Diagnosis**	76649	100	3	2-5	12549	16.0	5163	6.5	223	0.3	2644	3.2
**Respiratory, Any**	72997	94.9	3	2-5	11843	15.9	4970	6.6	209	0.3	2488	3.1
Influenza with Other Respiratory Manifestations	43125	56.1	2	1-4	3678	8.3	900	2.0	85	0.2	474	1.0
Pneumonia	28026	36.3	4	2-7	7330	25.6	3537	12.2	117	0.4	1738	5.7
Respiratory Failure	20143	26.6	5	3-8	8076	38.7	4274	20.2	103	0.5	1951	8.7
COPD Exacerbation	11081	14.7	4	2-6	2073	18.0	748	6.3	15	0.1	253	2.1
Asthma Exacerbation	7107	8.9	3	2-5	936	13	320	4.3	13	0.2	46	0.6
Other Acute Lower Respiratory Tract Disease	6473	8.8	3	2-6	974	14.6	382	6	22	0.3	143	1.9
Acute Upper Respiratory Infection	1393	1.7	2	1-3	100	7.1	19	1.3	0	0	5	0.3
Pulmonary Collapse	1269	1.6	4	2-7	237	17.9	93	7.2	3	0.2	39	2.9
Acute Respiratory Distress Syndrome	581	0.7	5	2-10	256	44.2	176	30.8	24	4.3	84	14.3
Pneumothorax	167	0.2	9	3-18	106	63.5	83	50.0	4	2.4	48	28.7
Mediastinitis	12	0.01	12	6-20	10	83.3	6	50.0	1	8.3	0	0

[a]Numbers are unweighted values, and percentages are weighted values. Acute diagnosis categories are not mutually exclusive unless otherwise stated.

© 2020 Chow EJ et al. *JAMA Network Open.*

eFigure 1. Venn Diagram of Acute Respiratory and Nonrespiratory Diagnoses, FluSurv-NET, United States, 2010-2018

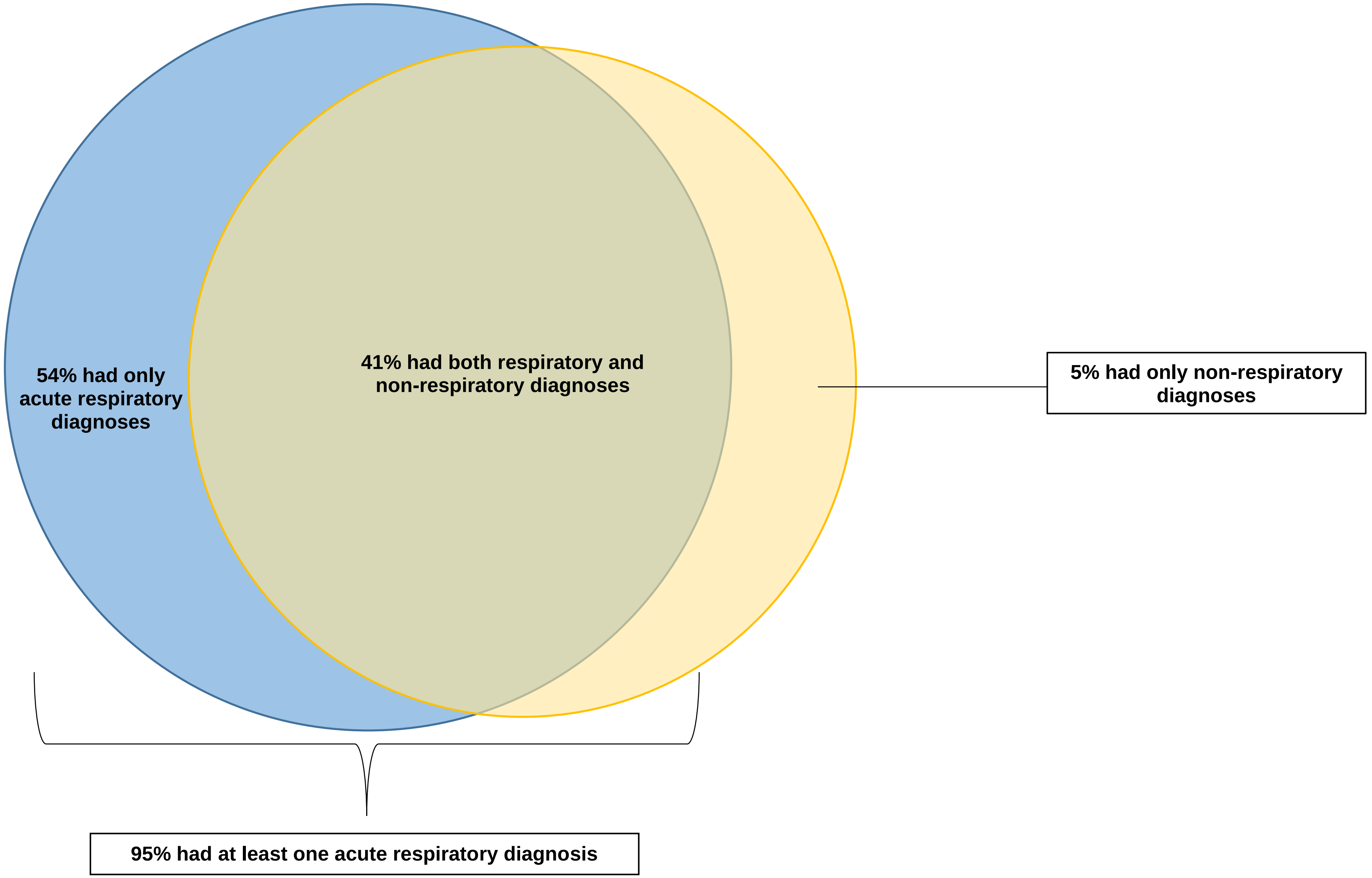

© 2020 Chow EJ et al. *JAMA Network Open.*

eFigure 2. *ICD* Code–Based Identification of Influenza and Related Diagnoses Among Adult Patients Hospitalized with Influenza, FluSurv-NET, United States, 2010-2018

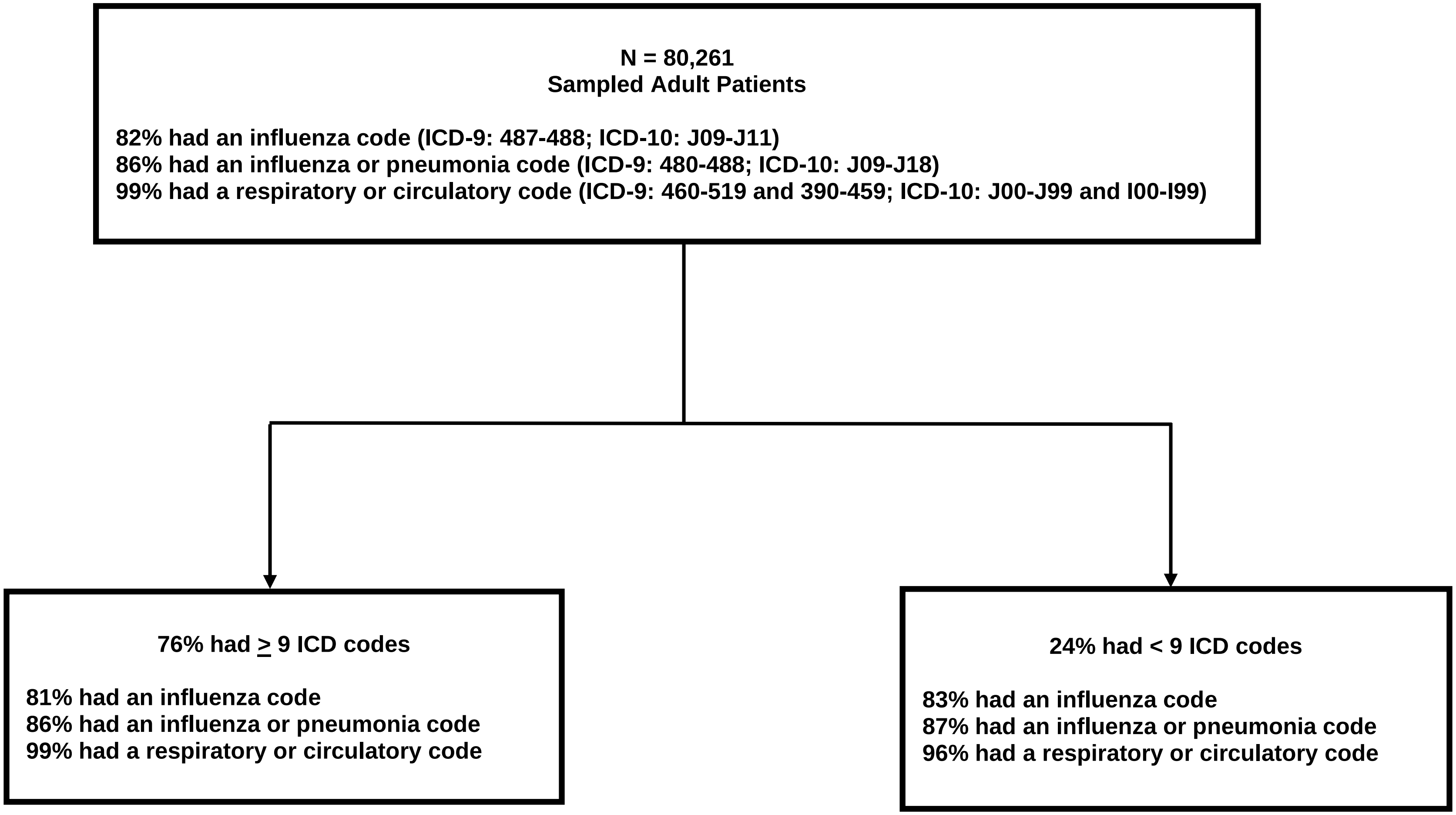

© 2020 Chow EJ et al. *JAMA Network Open.*
